# Hydrothermal Synthesis of High-Performance ZnO Nanorods for Enhanced Photocatalytic Degradation of Organic Pollutants

**DOI:** 10.1021/acsomega.6c01314

**Published:** 2026-04-14

**Authors:** Gonçalves J. Marrenjo, Paulo H. H. Nunes, Karen K. L. Augusto, Jéssica C. de Almeida, Caue Ribeiro, Antônio O. T. Patrocínio, Osmando F. Lopes

**Affiliations:** † Laboratoty of Photochemistry and Materials Science, Institute of Chemistry, 28119 Federal University of Uberlândia, Avenida João Naves de Avila, 2121, Uberlândia, Minas Gerais 34800-902, Brazil; ‡ Department of Natural Sciences, Save University, FPLM Avenida FPLM, 111, Massinga, Inhambane +25829371110, Mozambique; § Centro de Excelência em Hidrogênio e Tecnologias Energéticas Sustentáveis (CEHTES), Federal University of Goias, Goiânia, Goiás 74690-631, Brazil; ∥ EMBRAPA Instrumentação, Rua XV de Novembro, 1452, São Carlos, São Paulo 13560-970, Brazil

## Abstract

Excessive production and indiscriminate use of organic pollutants have caused severe environmental imbalances. Heterogeneous photocatalysis has emerged as a promising approach for the degradation of these contaminants. However, the development of photocatalysts with high activity and stability remains a key challenge. This work describes the hydrothermal synthesis of ZnO-based photocatalysts, carried out at different temperatures (25, 100, 150, and 200 °C), followed by an evaluation of their performance as photocatalysts in the degradation of organic pollutants (methylene blue, rhodamine B, amiloride and ciprofloxacin). ZnO samples exhibited rod-like morphology, especially in the samples synthesized at 100 °C. The ZnO-100 sample showed greater efficiency in the photodegradation of all organic pollutants. The enhanced performance can be attributed to its highest specific surface area and phase mixture between ZnO and Zn­(OH)_2_. The phase transition from zinc hydroxide to ZnO may have influenced the formation of a morphology that enhanced photocatalytic activity. Fluorescence analysis using terephthalic acid (TA) as a probe for OH^•^, combined with radical scavenging experiments, revealed that O_2_^–•^ play the dominant role in the photocatalytic degradation process, indicating an indirect reaction mechanism. This mechanism is consistent with the observed nonselectivity of the ZnO samples in removing pollutants from different chemical classes. The ZnO sample exhibited good stability over four consecutive reuse cycles and maintained high photocatalytic activity across a broad pH range (4–9). Overall, the hydrothermal method proved effective for producing ZnO nanorods with high photocatalytic performance for organic pollutant degradation.

## Graphical abstract

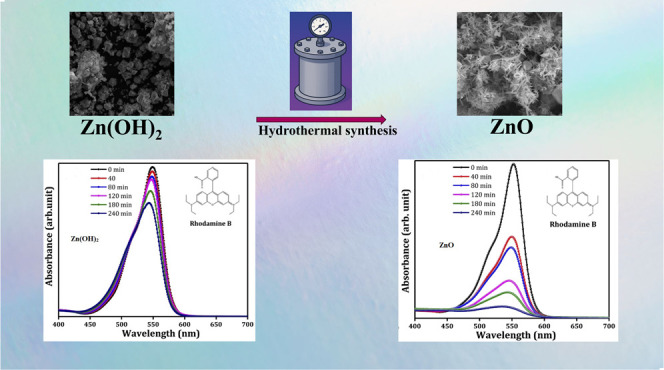

## 1 Introduction

Industrial sectors such as textiles, petrochemicals, pharmaceuticals, and agro-industries are among the major sources of organic pollutants released into aquatic environments, often without adequate treatment. The persistence of these contaminants in water bodies poses serious environmental concerns due to their toxicity, bioaccumulation potential, and resistance to natural degradation processes.^1–3^ Therefore, the development of efficient and sustainable technologies for wastewater treatment remains a critical challenge.^4–7^

Heterogeneous photocatalysis has emerged as a promising and environmentally friendly approach for the degradation of organic pollutants, as it enables the mineralization of contaminants into less harmful products under mild operating conditions. In this context, semiconductor-based photocatalysts have been extensively investigated due to their ability to generate highly reactive species under light irradiation, promoting the oxidative degradation of organic compounds.^8–10^

Among the various semiconductor materials, ZnO has attracted considerable attention owing to its intrinsic n-type conductivity, wide band gap of approximately 3.2 eV, low toxicity, chemical stability, and low production cost.^11–13^ In addition, ZnO can be synthesized through relatively simple and well-controlled methods, making it attractive for large-scale applications, for example continuous-flow hydrothermal synthesis, allowing for high-throughput production with consistent morphology and crystallinity.^14–19^ Several studies have demonstrated the effectiveness of ZnO as a photocatalyst for the degradation of organic pollutants under UV irradiation. More recently, advanced ZnO architectures, including hierarchical morphologies such as nanoflowers,^20^ pompon-like structures,^21^ and three-dimensional assemblies,^22^ have been reported to enhance photocatalytic efficiency by increasing surface area, improving light harvesting through multiple scattering, and facilitating charge separation.^23^ However, despite these advantages, ZnO still presents important limitations, such as a high electron–hole recombination rate and restricted operational stability, which can compromise its long-term performance.^24,25^

While numerous strategies have been proposed to improve ZnO photocatalytic efficiency, including doping and the formation of composite materials, comparatively less attention has been devoted to understanding how synthesis parameters influence the intrinsic properties of ZnO and their direct impact on photocatalytic behavior.^26^ In hydrothermal synthesis, temperature plays a crucial role in governing dissolution–reprecipitation processes, phase transformation from Zn­(OH)_2_ intermediates to ZnO, and anisotropic crystal growth along the *c*-axis of the wurtzite structure. Under specific conditions, incomplete conversion of Zn­(OH)_2_ can lead to phase mixtures, which may form ZnO/Zn­(OH)_2_ heterointerfaces capable of influencing charge separation and surface reactivity.^27^ These factors strongly affect morphology, crystallinity, charge carrier dynamics, and surface reactivity, highlighting the importance of systematic studies correlating synthesis conditions with structure–property–performance relationships.^28,29^

Furthermore, most photocatalytic studies employing ZnO focus on the degradation of a single model pollutant, typically a dye, which does not adequately represent the chemical complexity of real wastewater. Organic contaminants differ substantially in molecular structure, charge, functional groups, and degradation pathways, which directly influence adsorption behavior and reactivity toward photogenerated species. As a result, photocatalysts evaluated using only one class of pollutants may exhibit limited applicability when exposed to chemically diverse contaminants.^30,31^

In this study, ZnO materials were synthesized via a hydrothermal route at different temperatures, and the influence of synthesis temperature on their physicochemical properties, morphology, and photocatalytic behavior was systematically investigated. Beyond conventional single-pollutant evaluations, the versatility of the ZnO photocatalyst was assessed through the degradation of four organic contaminants with markedly different chemical characteristics, including two dyes (methylene blue (MB) and rhodamine B (RhB)) and two pharmaceutical compounds (amiloride and ciprofloxacin), under UV irradiation. The photocatalytic mechanism was elucidated through indirect detection of hydroxyl radicals using terephthalic acid as a fluorescence probe and by radical scavenging experiments employing benzoquinone and *tert*-butanol to identify the dominant reactive species. In addition, the operational stability of ZnO was evaluated through consecutive reuse cycles, and the effect of solution pH on pollutant degradation was examined, providing comprehensive insight into the robustness and practical applicability of the synthesized photocatalyst.

## 2 Materials and Methods

### 2.1 Synthesis of ZnO Samples

The ZnO precursor (denoted as Prec) was obtained by a precipitation method. Initially, a 1.0 mol L^–1^ HNO_3_ solution (Vetec, 65%) was added dropwise to a 0.025 mol L^–1^ aqueous solution of Zn­(NO_3_)_2_·6H_2_O (Êxodo Científica, 90%) prepared in 200 mL of deionized water under continuous stirring, until the pH reached 3, ensuring complete dissolution of the zinc salt. Subsequently, the pH of the solution was adjusted to 8 by the addition of NH_4_OH solution (Isofar, 24%).^18,32^ This step promotes controlled nucleation while limiting excessive particle growth.^33^ The suspension was maintained under constant stirring for 24 h, leading to the formation of a white precipitate. The resulting solid was collected by centrifugation, thoroughly washed with deionized water to remove residual ions, and dried in an oven at 80 °C for 24 h. The obtained material was labeled as Prec.

For the synthesis of ZnO materials, 0.3 g of the Prec sample was dispersed in 100 mL of deionized water and subjected to hydrothermal treatment at different temperatures (100, 150, and 200 °C) in a hydrothermal reactor under stirring for 2 h. After each treatment, the supernatant was discarded, and the solid was recovered by centrifugation, washed with deionized water, and dried at 60 °C for 24 h. The samples obtained after hydrothermal treatment were labeled according to the synthesis temperature as ZnO-100, ZnO-150, and ZnO-200, respectively, while the untreated precursor was referred to as “Prec”.

### 2.2 Characterizations of ZnO Samples

The structural properties of the as-synthesized ZnO samples were examined by X-ray powder diffraction (XRD). The diffraction patterns were obtained using a Shimadzu Lab-X XRD 6000 diffractometer, equipped with Cu Kα radiation (λ = 1.5406 Å), operating at 40 kV and 30 mA. Scanning was carried out in the range of the 2θ from 10 to 70°, with a scan rate of 1° min^–1^. The Scherrer equation was used to determine the average crystallite size from the XRD data.^32,34,35^ The infrared spectra were collected using a PerkinElmer Frontier Fourier transform infrared spectrometer (FTIR) in attenuated total reflectance (ATR) mode with a diamond crystal. Each spectrum was obtained with 32 scans and a resolution of 4 cm^–1^, in the range 220 cm^–1^ to 4000 cm^–1^. This technique made it possible to investigate the molecular vibrations of a substance through the absorption of infrared radiation at different wavelengths, making it possible to identify the functional groups on the surface of the sample. It was also crucial for verifying the contamination of the material during its synthesis. Raman spectroscopy analysis was performed at HORIBA LabRAM HR Evolution, with laser excitation of 532 nm, potential filter 3.2%, 600 lines mm^–1^ and 6 scans accumulation. The reflectance and absorption coefficient measurements were obtained from diffuse reflectance spectroscopy (DRS) with a Shimadzu UV-2600 UV–vis spectrophotometer. The absorbance of the materials studied was observed in the visible region between 400 and 700 nm. The direct energy of the band gap (E_g_) was calculated by the Tauc plot.^36^

The morphology of the samples was examined by scanning electron microscopy (SEM) using a Tescan VEGA 3 LMU microscope, which allowed for the morphological evaluation of the obtained samples. Transmission electron microscopy (TEM) was also conducted using a FEI TECNAI G2 F20. To prepare samples for TEM analysis, a drop of the colloidal suspension of each sample was deposited onto carbon-coated copper grids and dried under ambient conditions.

The specific surface area (SSA) of the samples was estimated by N_2_ physisorption analysis at 77 K in the Micrometrics ASAP 2000 equipment (Norcross, Georgia, USA) applying B.E.T. (Brunauer–Emmett–Teller) model. Prior to the analysis, the samples were pretreated (degasification) by heating at 80 °C under vacuum until reaching a degassing pressure lower than 10 μmHg. Thermogravimetric analysis (TGA) was carried out in a temperature range of 10 to 600 °C, at a heating rate of 10 °C min^–1^, under synthetic air atmosphere at a flow rate of 50 mL min^–1^, using Shimadzu DTG-60H equipment. X-ray photoelectron spectroscopy (XPS) analysis was performed using an Scienta-Omicron hemispherical analyzer. The spectra were acquired using a monochromatic Al Kα radiation source (*hv* = 1486.7 eV) operated at 15 kV. High-resolution spectra for C 1s, O 1s, and Zn 2p regions were recorded with a pass energy of 30 eV and a step size of 0.05 eV. The survey spectra were collected using a pass energy of 50 eV. All binding energies were calibrated using the adventitious C 1s peak at 284.8 eV as an internal reference. Data processing and peak fitting were conducted employing Shirley or Tougaard backgrounds and Gaussian–Lorentzian GL(30) line shapes.

### 2.3 Photocatalytic Activity and Stability Evaluation

The photocatalytic performance of the ZnO samples for organic pollutant degradation was evaluated under UV illumination for two dyes, MB and RhB, and two drugs, amiloride (AML) and ciprofloxacin (CIP). A suspension of 25 mg of ZnO was dispersed in 50 mL of each pollutant solution, with concentrations of 10 mg L^–1^ for MB, 5 mg L^–1^ for RhB, and 10 mg L^–1^ for CIP and AML. The suspensions were kept in the dark for 12 h to ensure the complete adsorption–desorption equilibrium of the contaminant. Then, they were placed in a photoreactor at a controlled temperature (18 °C), under UVC radiation (Philips TUV lamps, 15 W; maximum intensity at 254 nm and average irradiance of 40 W m^–2^), Figure S1. The organic pollutant concentration was monitored in a UV–vis spectrophotometer (Shimadzu UV-1650PC) at regular time intervals, photodegradation was quantified by the reduction in pollutant concentration at their respective absorption wavelengths. All tests were conducted in triplicate.

The stability of ZnO was evaluated through four consecutive photocatalytic degradation cycles, with catalyst recovery, washing, and reuse after each cycle, while monitoring the photocatalytic efficiency throughout the repetitions.^37^ After the stability test, the material was characterized by XRD and SEM to assess possible structural and morphological changes.

Chemical oxygen demand (COD) measurements were performed after each cycle to monitor degradation of the pollutants. COD was determined using the dichromate oxidation method, following an adaptation of USEPA Method 410.4, with measurements carried out using a Hanna HI83099 COD and multiparameter photometer. Analytical-grade reagents were employed, including concentrated sulfuric acid (H_2_SO_4_), silver sulfate (Ag_2_SO_4_), potassium dichromate (K_2_Cr_2_O_7_), and mercury sulfate (HgSO_4_). A sulfuric acid-silver sulfate solution was prepared by dissolving Ag_2_SO_4_ in concentrated H_2_SO_4_ at a ratio of 5.5 g kg^–1^ and allowing the mixture to stand for 1–2 days to ensure complete dissolution. The potassium dichromate solution (0.167 mol L^–1^) was prepared by drying K_2_Cr_2_O_7_ at 103 °C for 2 h. COD digestion was performed in Hach tubes by sequential addition of 0.04 g of HgSO_4_, 2.5 mL of the sulfuric acid-silver sulfate solution, 0.3 mL of distilled water, 0.5 mL of potassium dichromate solution, and 2.0 mL of sample. The tubes were sealed, mixed, and digested at 150 °C for 2 h, then cooled to room temperature prior to spectrophotometric measurement.

### 2.4 Evaluation of Degradation Mechanism

To investigate the degradation mechanism of the ZnO samples, terephtalic acid was used as a probe for detecting photogenerated OH^•^ by means of fluorescence.^38,39^ In all experiments, 50 mL of terephtalic acid solutions (5 × 10^–4^ mol L^–1^) were prepared in a diluted NaOH solution (2 × 10^–3^ mol L^–1^). Subsequently, the ZnO-based photocatalysts were suspended in the prepared solutions at a concentration of 100 mg L^–1^ and irradiated for 1 h by a xenon lamp (300 W), with the total irradiance set at 100 mW cm^–2^. Aliquots were taken from the irradiated solutions at set intervals of 15 min, which were then filtered and analyzed by a Horiba spectrofluorometer, model Fluoromax-4, at ambient temperature, at the 350 to 600 nm range and excitation wavelength of 315 nm, allowing for the assessment of the concentration of the generated 2-hydroxyterephtalyic acid byproduct, which has an emission peak at 425 nm. The general formation of the 2-hydroxyterephtalyic acid is depicted in Figure S2.

Radical scavenging experiments were carried out using 10 mL of RhB solution mixed with 5 mg of the ZnO suspension. *tert*-Butanol (1 μL) and benzoquinone (1 mg) were added separately as OH^•^ and O_2_^–•^ scavengers, respectively. The photocatalytic performance obtained in the presence of each scavenger was compared with that of a control experiment conducted in the absence of scavengers.^40^

## 3 Results and Discussion

### 3.1 Characterization of ZnO Samples

XRD analysis of the precursor synthesized at 25 °C showed characteristic peaks of the crystallographic phase corresponding to the Zn­(OH)_2_ phase (PDF 012-0479) and results are shown in Figure 1a.^32,41^ The formation of Zn­(OH)_2_ under this condition was expected, since similar studies have observed that the use of water as a solvent during the synthesis of ZnO under alkaline conditions and at room temperature can lead to its precipitation.^32,42,43^ On the other hand, treatment of the Prec at temperatures above 100 °C in the hydrothermal reactor resulted in the formation of a ZnO crystal structure, with indexed patterns in the hexagonal wurtzite phase (PDF 36-1451).^42,43^ It was not observed significant differences in the crystallite size of the ZnO samples (Table S1), indicating that the increase in temperature did not contribute to crystal size growth. In this case, the crystallite size growth appears to be more related to the kinetics of the process rather than thermodynamic factors.

**Figure 1:**
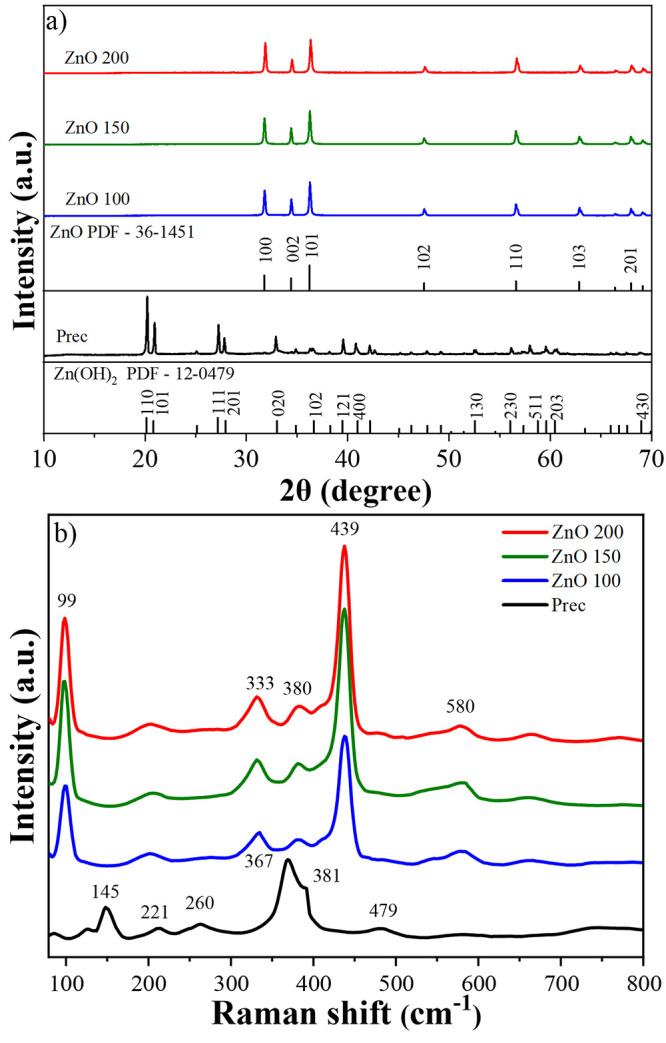
(a) XRD patterns and (b) Raman spectra of the ZnO powder samples.

The ZnO samples were characterized by Raman spectroscopy to gain deeper insight into their short-range structural order and local lattice features. The Raman spectrum of Prec sample reveals several well-defined peaks at 145, 221, 260, 367, 380 e 479 cm^–1^ (Figure 1b). These features are characteristic of zinc hydroxide, with the band at 145 cm^–1^ typically attributed to lattice vibrations involving Zn–OH bonds, while the peaks at 260 cm^–1^ and 367 cm^–1^ are associated with bending modes of the hydroxyl groups bonded to Zn. The peak at 479 cm^–1^ can be assigned to Zn–O stretching modes within the hydroxide structure.^32,44^ After hydrothermal treatment, all ZnO samples display Raman peaks at 99, 333, 380, and 439 cm^–1^, which are consistent with the wurtzite hexagonal structure of ZnO. The prominent peak at 99 cm^–1^ corresponds to the E_2_^low^ mode, related to the vibration of the Zn sublattice. The band at 333 cm^–1^ arises from a second-order Raman process 2 E_2_. The peak at 380 cm^–1^ corresponds to the A_1_ (TO) mode, while the intense band at 439 cm^–1^ is associated with the E_2_^high^ mode, which is indicative of high crystalline quality and is related to oxygen atom vibrations in the ZnO lattice.^39,45,46^

The FTIR spectra of Prec and ZnO samples obtained under different hydrothermal conditions, allowing the monitoring of structural and compositional evolution as a function of synthesis temperature Figure 2a. All samples exhibited a broad band in the 3400–3500 cm^–1^ region, attributed to the stretching vibrations of O–H bonds from water molecules adsorbed on the surface.^47–49^ The intensity of this band decreased significantly in the samples treated at 150 and 200 °C, indicating that higher temperatures favor water desorption and the elimination of surface hydroxyl groups. In the samples synthesized at 25 and 100 °C, bands between 1519 and 1624 cm^–1^ were observed, related to the bending vibrations of surface water molecules (H–O–H). Additionally, the material prepared at 25 °C exhibited additional bands in the range of 800 to 1500 cm^–1^, assigned to hydroxyl groups and Zn–OH stretching vibrations, confirming the predominance of the Zn­(OH)_2_ phase under this condition.^48–50^ Conversely, the samples treated at 100 °C, 150 °C, and 200 °C displayed characteristic bands between 450 and 500 cm^–1^, associated with the stretching vibrations of Zn–O bonds, evidencing the formation of the ZnO phase as observed by XRD and Raman analyses.^18,51^

**Figure 2:**
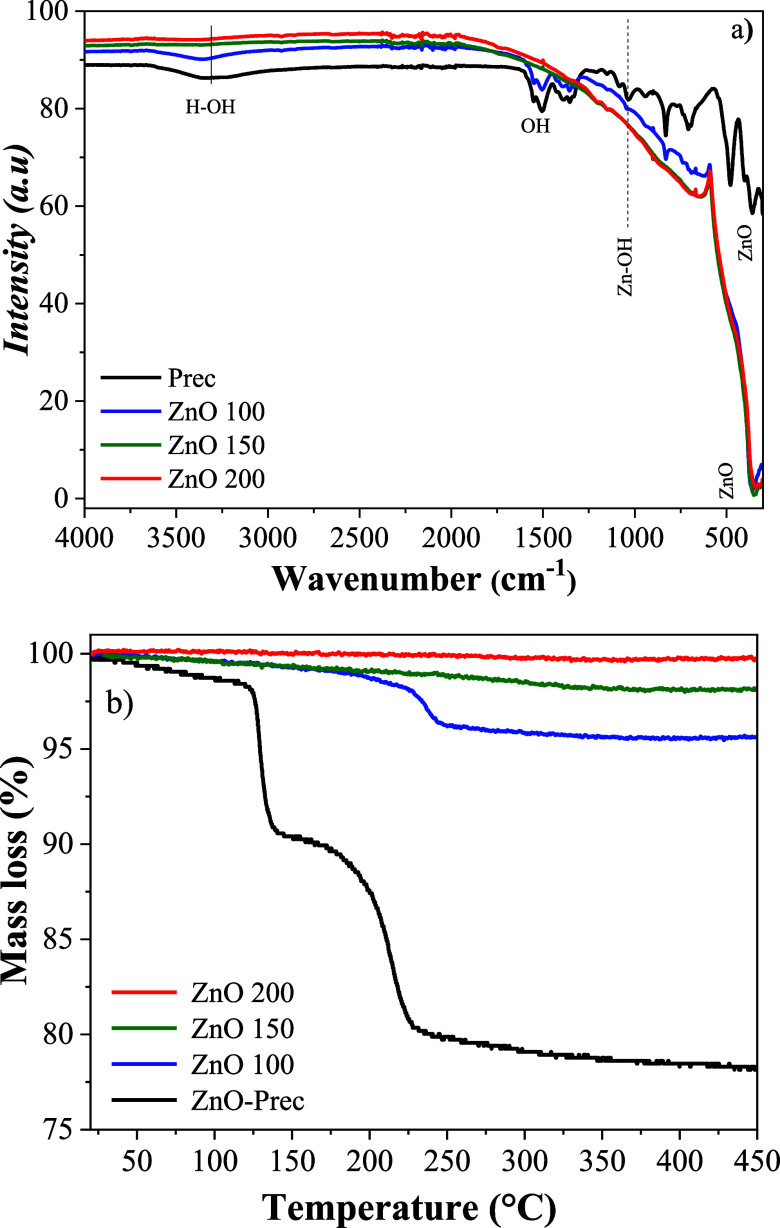
(a) FTIR spectra and (b) TGA curves of the synthesized ZnO powder samples.

TGA was carried out for the samples synthesized under different hydrothermal treatment conditions, as illustrated in the thermograms presented in Figure 2b. For the Prec sample obtained at 25 °C two distinct mass loss events were identified. The first event occurred around 100 °C, with a mass loss of approximately 10%. This loss is attributed to the removal of physically adsorbed water from the material’s surface.^52^ The second event was observed around 200 °C, also with a mass loss of approximately 10%. This mass loss can be associated with the thermal decomposition of Zn­(OH)_2_, promoting its conversion to ZnO. During this stage, both the adsorbed and the structurally bound water are released, characterizing a dihydroxylation process accompanied by structural reorganization of the material.^51,53^ In the ZnO sample treated at 100 °C, it can be observed the same profile, however the mass loss is smaller, around 5%. This behavior suggests that although part of the adsorbed water was removed during the hydrothermal process, a small amount of the Zn­(OH)_2_ phase remains in the material’s structure, indicating that the conversion of Zn­(OH)_2_ into ZnO was not complete during the hydrothermal step.^54^ The absence of Zn­(OH)_2_ peaks in the XRD patterns of the ZnO 100 sample does not necessarily indicate the complete disappearance of this phase. XRD mainly detects well-crystallized phases with long-range structural order. Therefore, if Zn­(OH)_2_ remains in small amounts or as poorly crystalline domains, its diffraction signal may fall below the detection limit of the technique. In this context, the mass loss observed around 200 °C in the TGA profile of the ZnO 100 sample provides indirect evidence of residual Zn­(OH)_2_ through its dehydroxylation to ZnO.

In contrast, the samples treated at 150 and 200 °C showed no significant mass loss events throughout the heating process. This result indicates that, under these conditions, the complete removal of chemisorbed hydroxyl species (OH^–^) occurred, favoring the formation of pure crystalline ZnO.^18,53,55^ The absence of hydroxyl-related bands in the FTIR spectra of these samples reinforces this observation, confirming the formation of pure and crystalline wurtzite-type ZnO. This phase is thermodynamically stable at high temperatures (above 700 °C) and is characteristic of well-crystallized ZnO.^53,54^ Therefore, the TGA results, combined with complementary FTIR and XRD analyses, indicate that hydrothermal treatments at 150 and 200 °C favor the formation of high-purity zinc oxides, with complete elimination of intermediate hydroxylated phases and stabilization of the wurtzite phase.

The sample’s morphologies were examined by SEM and TEM images, and the results are shown in Figure 3. SEM analyses of the ZnO precursor sample treated at 25 °C showed an undefined morphology, with diffuse edges block-like shapes, which is associated with the predominance of the Zn­(OH)_2_ phase (Figure 3a).^45^ The hydrothermal treatment promoted the formation of rod-shaped particles (Figure 3b), with a well-defined morphology typical of ZnO, attributed to the gradual removal of hydroxyl groups.^18,56^ The increase in temperature induces the phase transformation of Zn­(OH)_2_ into ZnO, promoting its solubilization and subsequent reprecipitation, thereby determining the morphological evolution of the material. At lower temperatures, the resulting morphology is irregular, whereas higher temperatures favor the formation of compact and aggregated structures.^51,56^ The samples treated at 150 and 200 °C exhibited minimal morphological changes, limited mainly to a slight reduction in rod length (Figure 3c,d). This stabilization of the rod morphology was possibly caused by the equilibrium in crystal growth and the almost complete removal of the hydroxyl groups. Thus, the hydrothermal treatment efficiently led to the morphological transition from the Zn­(OH)_2_ to the stable crystalline phase of ZnO. SEM images also allowed the evaluation of the morphology and surface dimensions of the ZnO particles through statistical analysis based on size distribution histograms (Table S2). Zn­(OH)_2_ exhibited larger and thicker particles, with an average length of 14.6 μm and width of 14.1 μm, characteristic of a poorly crystalline precursor. After conversion to ZnO, a progressive reduction in apparent length and changes in surface morphology were observed: ZnO 100 (3.4 × 0.5 μm), ZnO 150 (3.4 × 0.6 μm), and ZnO 200 (4.0 × 0.8 μm). These results indicate structural reorganization and increased surface compaction of the particles with increasing temperature, reflecting the direct effect of thermal treatment on the material’s morphology.

**Figure 3:**
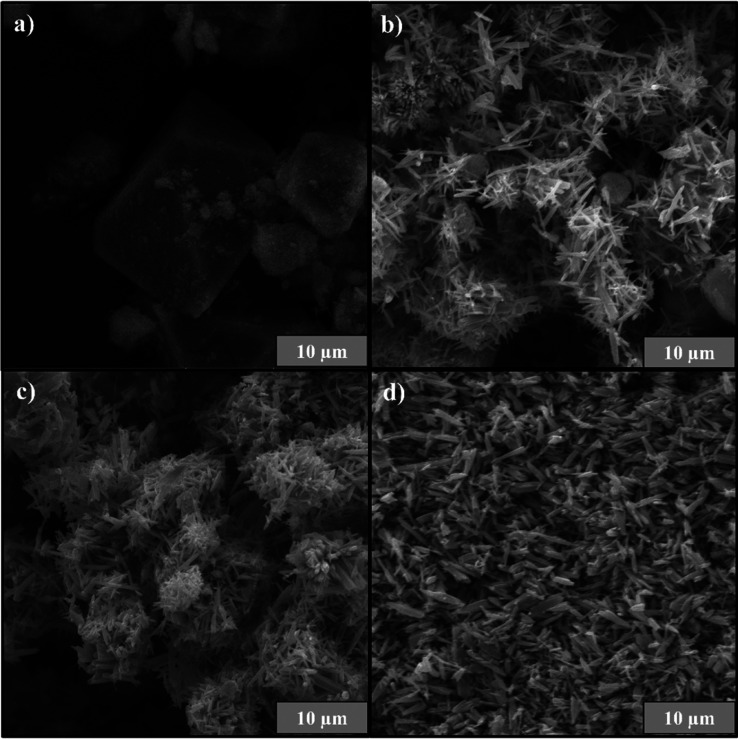
SEM images for (a) Prec sample and ZnO samples at different temperatures: (b) 100 °C, (c) 150 °C, and (d) 200 °C.

The morphological differences observed in ZnO synthesized at different temperatures are mainly associated with the temperature-dependent crystallization and phase transformation during the hydrothermal process.^57,58^ At 100 °C, the relatively low thermal energy is insufficient to promote complete dehydration and recrystallization of the precursor Zn­(OH)_2_ phase into ZnO. As a result, the ZnO-100 sample exhibits heterogeneous morphologies, including rod-like structures combined with plate-like and poorly defined features, which are characteristic of residual or partially transformed Zn­(OH)_2_.

The TEM was employed to obtain images capable of revealing structural details at the nanometric scale, allowing the analysis of individual particle morphology and the investigation of interfacial regions between ZnO nanorods (Figure 4a,b). After 2 h of thermal treatment, all samples retained their characteristic nanorod-like morphology. These morphological changes suggest that thermal treatment promotes anisotropic growth, likely due to a reduction in hydroxide ion surface enrichment, which in turn influences nucleation and crystal growth rates. HRTEM images were acquired to further investigate the crystallographic structure of the samples at the nanoscale (Figure 4c,d). For the ZnO 100 sample, clear lattice fringes with an interplanar spacing of approximately 0.28 nm were observed, which can be assigned to the (100) plane of hexagonal wurtzite ZnO. Interestingly, additional regions displaying lattice spacing close to 0.32 nm were also detected, which can be attributed to the (111) plane of Zn­(OH)_2_. The coexistence of these lattice fringes suggests the presence of localized hydroxide domains within the ZnO structure, indicating that the hydrothermal treatment at 100 °C does not promote complete conversion of the precursor. In contrast, the ZnO 200 sample mainly exhibits lattice fringes corresponding to ZnO, these observations provide direct nanoscale evidence supporting the phase transition from Zn­(OH)_2_ to ZnO during the hydrothermal process and corroborate the results obtained from TGA and FTIR analyses.

**Figure 4:**
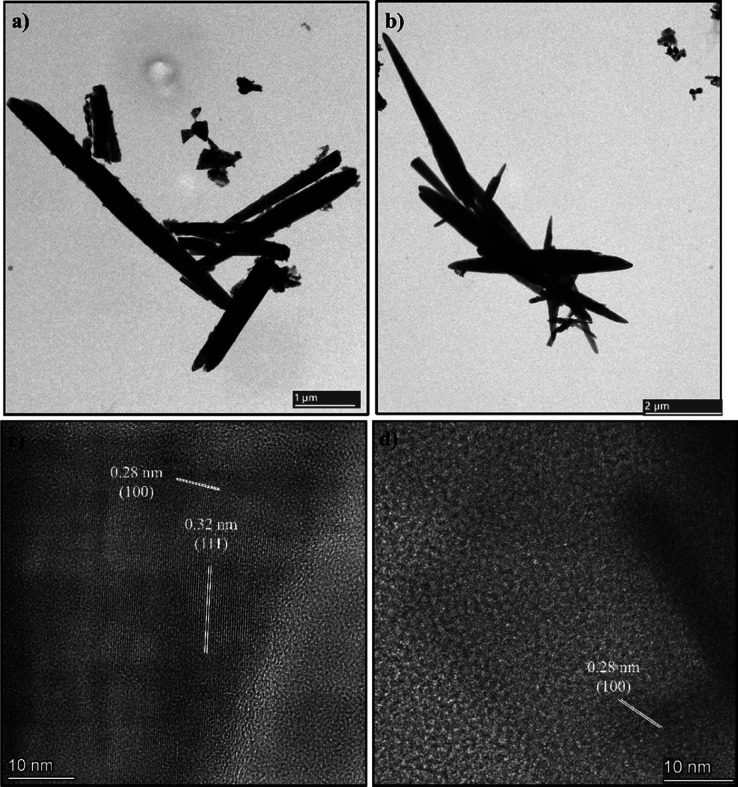
TEM images for (a) ZnO 100 and (b) ZnO 200 samples. HRTEM images for (c) ZnO 100 and (d) ZnO 200 samples.

The SSA analyses, obtained by nitrogen physisorption (Table 1), revealed that the samples treated at 25 °C, 150 °C, and 200 °C exhibited values <1 m^2^/g. These low values can be attributed to the formation of poorly developed morphologies, such as diffuse blocks or short rods, which limit the exposure of active surface sites.^53,54^ In contrast, the sample treated at 100 °C showed a surface area of 5 m^2^ g^–1^, at least 5 times higher than the other samples. This result indicates that the hydrothermal treatment at this temperature favored the growth of more elongated particles with enhanced surface exposure, it is expected, since morphological variations directly affect the SSA of ZnO.^48,50,53,59,60^ Therefore, the treatment at 100 °C stood out as the most effective condition for obtaining a morphology with greater surface accessibility.

**Table 1:** SSA of the ZnO Samples Showed That ZnO-100 Had a Relatively Higher Value Compared to the Other Samples

samples	SSA (m^2^ g^–1^)
Prec	<1
ZnO 100	5
ZnO 150	<1
ZnO 200	<1

The optical properties of the Prec and ZnO samples treated at different temperatures was evaluated by DRS. The DRS spectra (Figure S3) shows that all samples exhibit a strong absorbance around 380 nm.^53,61^ Therefore, hydrothermal synthesis resulted in samples that were photosensitive in the UV–visible region, reinforcing evidence that ZnO has excellent optical properties, particularly in the UV–visible range. Previous studies confirm that these optical properties make ZnO a promising material for various photocatalytic applications.^61,62^ DRS measurements presented in Figure S4 were used to estimate the optical band gap values by applying the Kubelka–Munk function. The ZnO samples synthesized at different temperatures exhibited direct band gap values of around 3.1–3.2 eV.^53,63,64^ These results indicate that increasing the synthesis temperature up to 200 °C does not induce significant changes in the electronic structure of the ZnO samples. Although temperatures above 100 °C promote recrystallization, they do not induce substantial changes in the electronic structure that could affect the band gap, demonstrating that the wurtzite phase of ZnO remains stable.^53,65^

To further investigate surface chemical states and elemental composition of the ZnO samples, XPS analysis was performed (Figure 5). The O 1s spectra for both ZnO-100 (Figure 5a) and ZnO-200 (Figure 5c) samples were deconvoluted into three distinct oxygen environments: lattice oxygen (O_latt_ at ∼530.0 eV), oxygen in hydroxide or deficient regions (O_OH_/O_def_ at ∼531.3 eV), and surface adsorbed species (O_ads_ at ∼532.3 eV).^66–68^ A striking difference was observed in the relative contribution of these species. The ZnO-100 sample exhibited an O_OH_/O_def_ content of 39.7 at. %, significantly higher than the 23.8 at. % observed for the ZnO-200 sample. This enrichment in surface hydroxyls and oxygen-related defects is a direct consequence of the lower hydrothermal temperature of 100 °C, which prevents complete dehydroxylation.^66–68^ The Zn 2p spectra in Figure 5b,d corroborates these findings and provides a deeper understanding of the surface composition. For the ZnO-200 sample, the Zn 2p 3/2 peak is sharp and symmetric, assigned entirely to the Zn^2+^ state within the stoichiometric wurtzite lattice (Zn–O).^66–68^ In contrast, the ZnO-100 sample requires deconvolution into two environments: a lattice Zn–O component at 1021 eV, representing 21.9 at. %, and a Zn–OH contribution at 1022 eV, representing 78.1 at. % of the surface zinc signal.^66–68^ The predominance of the Zn–OH species in the 100 °C sample is highly consistent with HRTEM image and the mass loss observed in the TGA analysis, which was attributed to the dehydroxylation of residual Zn­(OH)_2_ phase. This high concentration of surface hydroxyls plays a pivotal role in enhancing photocatalytic reactivity, as these species serve as active sites for pollutant adsorption and facilitate the generation of reactive hydroxyl radicals, contributing in indirect reaction mechanisms.^66–68^ These findings confirm that the hydrothermal treatment at 100 °C preserves a crucial phase mixture between ZnO and Zn­(OH)_2_ at the catalyst surface, providing a higher potential for chemical interaction compared to the more crystalline ZnO 200 surface.

**Figure 5:**
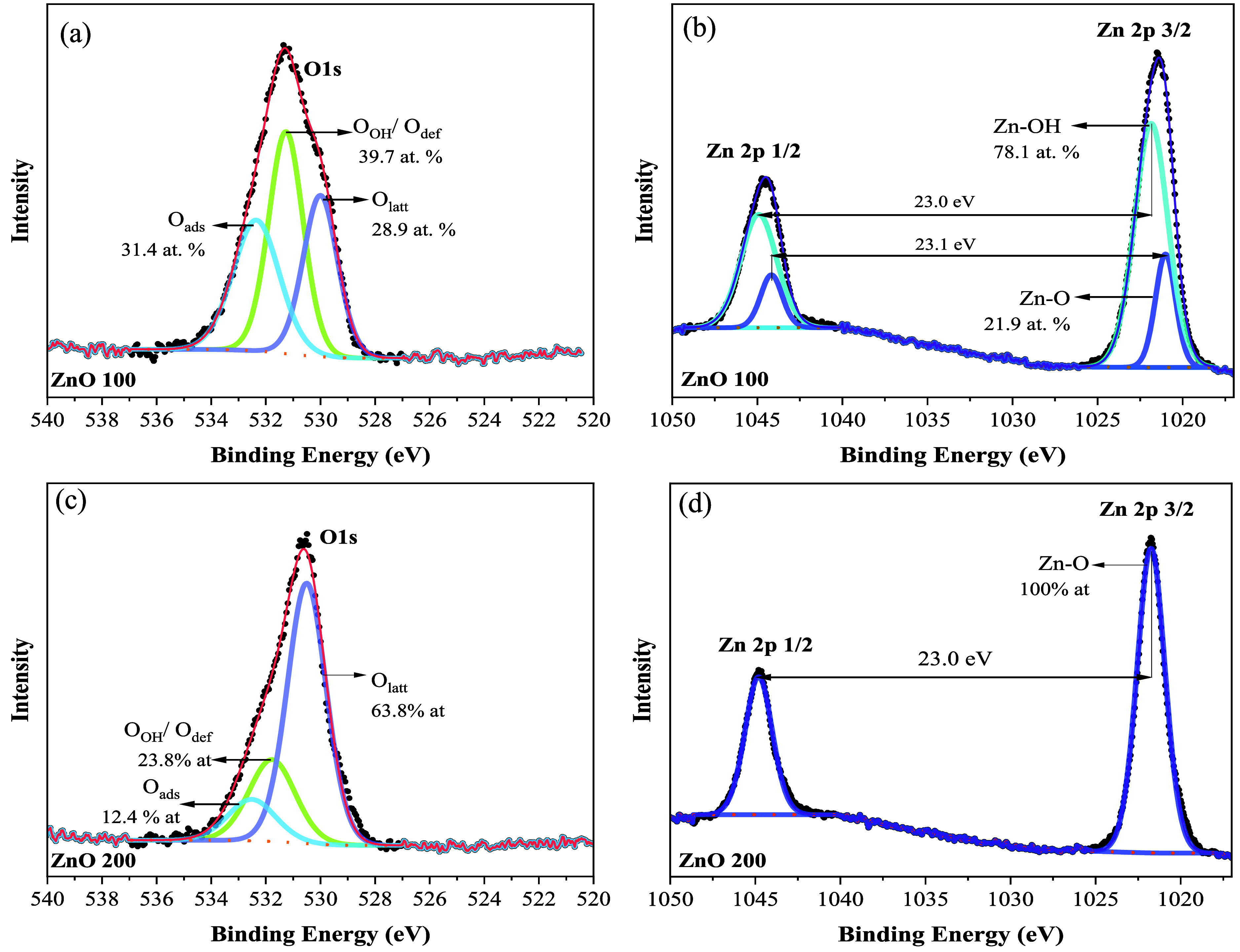
High-resolution XPS spectra of ZnO 200 and ZnO 100 samples for (a,c) O 1s spectra and (b,d) Zn 2p spectra.

### 3.2 Photocatalytic Performance of ZnO Samples

The photocatalytic activity of ZnO samples synthesized under different thermal conditions was evaluated through the degradation of four organic pollutants: two dyes (MB and RhB) and two drugs (AML and CIP). AML and CIP were selected as representative pharmaceutical pollutants due to their high environmental relevance and chemical persistence in aquatic systems. Ciprofloxacin is among the most frequently detected antibiotics in wastewater effluents and surface waters, largely due to its extensive use and structural complexity, which leads to slow degradation and the formation of stable transformation products.^69–71^ Amiloride, an emerging pharmaceutical contaminant, exhibits limited biodegradability associated with its heterocyclic framework and multiple ionizable functional groups.^72^ The inclusion of these compounds enables evaluation of photocatalytic performance toward pharmaceuticals with distinct molecular structures, charge states, and degradation pathways, providing a more realistic assessment compared to conventional dye-based models.

All samples demonstrated the ability to promote the photodegradation of the investigated pollutants, although with different efficiencies. The versatility of these materials to promote the degradation of different compounds was assessed through photocatalytic experiments, with the corresponding degradation kinetics illustrated in Figure 6. The variation in activity was evidenced by the gradual decrease in absorbance in the UV–vis spectra over the irradiation period. It can be observed that all samples were photoactive to promote the degradation of four organic pollutants. The ZnO sample treated at 100 °C exhibited the highest photocatalytic activity for all organic pollutants evaluated. This sample promoted the complete removal of all organic pollutant after 120 to 180 min of UV irradiation (Figure 7).^49,50,65,73^

**Figure 6:**
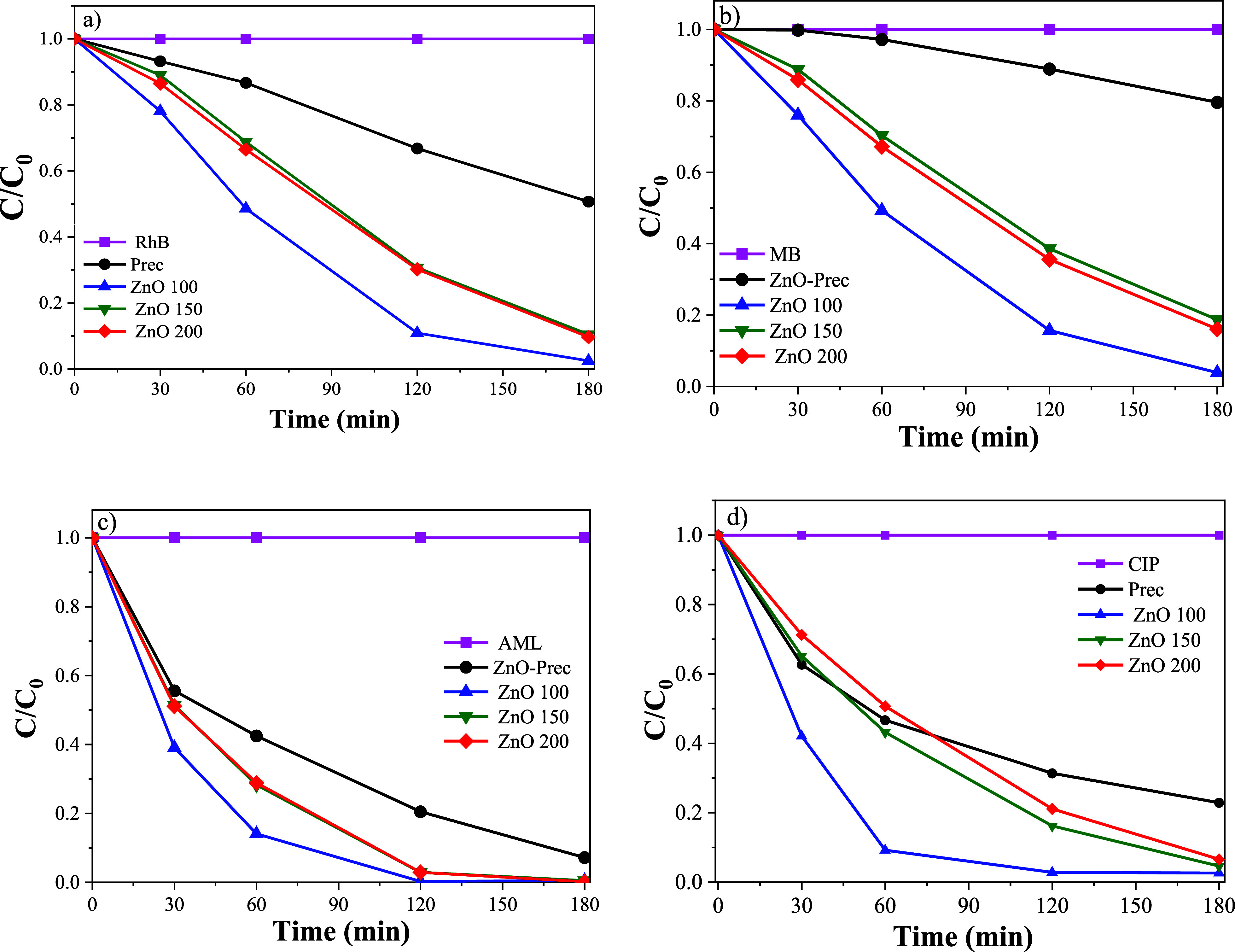
Photodegradation kinetics for (A) RhB, (B) MB, (C) AML, and (D) CIP under UV irradiation, catalyzed by Prec and ZnO samples synthesized at different temperatures (100, 150, and 200 °C).

**Figure 7:**
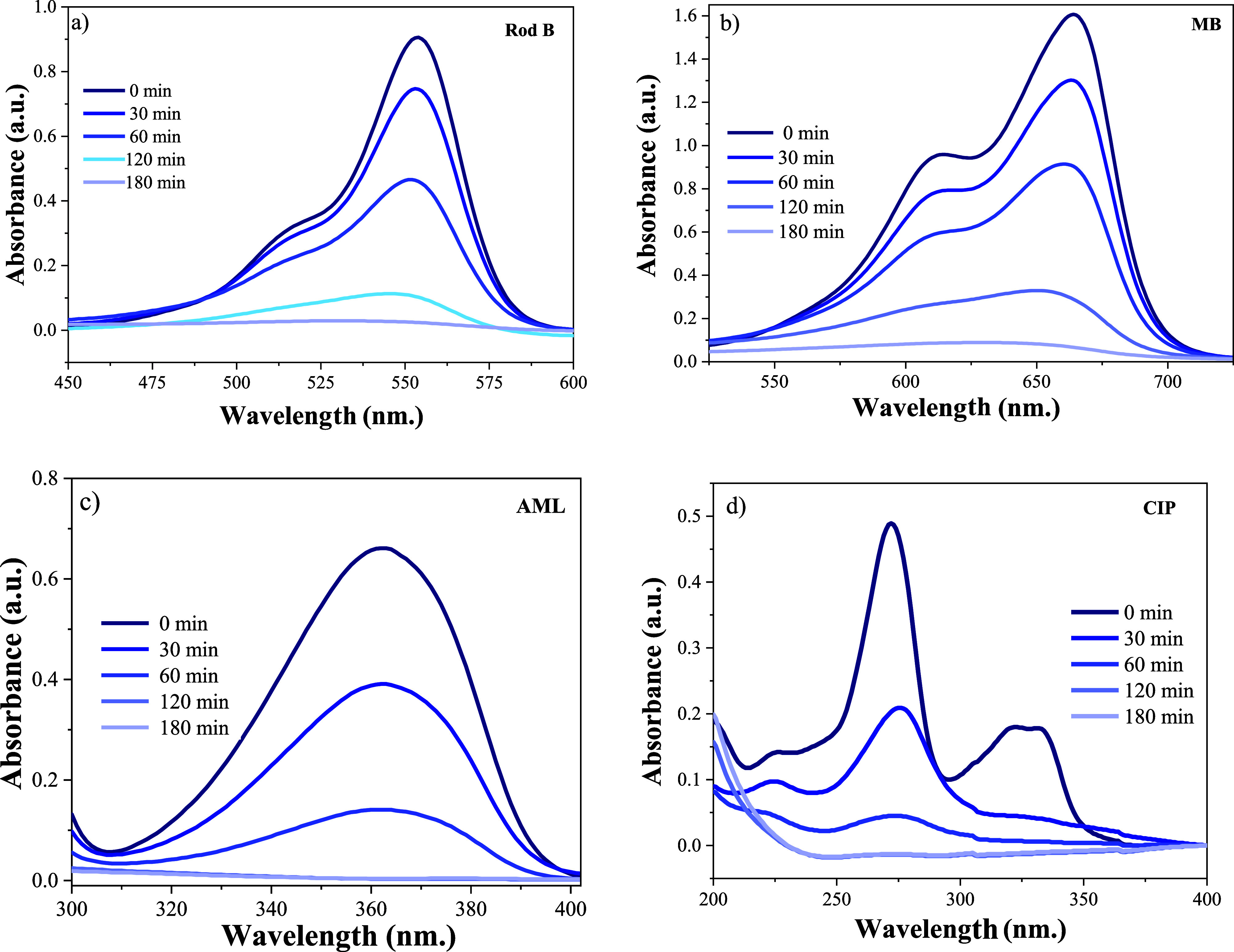
UV–vis absorption spectra of the organic pollutant degradation by ZnO 100, in (A) RhB, (B) MB, (C) AML, and (D) CIP.

The trend of lower photoactivity observed for the Zn­(OH)_2_ sample across all pollutants could be mainly associated with its inadequate electronic properties.^39,45,65,74^ Compared to the materials containing the ZnO phase, the unfavorable redox potentials in the valence and conduction bands, along with the reduced SSA are factors that significantly limit the photocatalytic potential of the Zn­(OH)_2_ sample.^39,45,48,65^ Although the samples treated at 150 and 200 °C also exhibit low SSA, the formation of the ZnO phase was crucial for the high photocatalytic activity of the pollutants, which may be related to its purity and the combination of more favorable electronic properties compared to the Zn­(OH)_2_ materials, allowing for more efficient adsorption of contaminants in aqueous medium and more effective degradation of these compounds.

This result indicates that the hydrothermal treatment of the Zn­(OH)_2_ sample induced a phase transition to ZnO, enhancing its photocatalytic performance. Conversely, the presence of a hydroxide phase along with oxides significantly increased the material’s active sites, as indicated by TGA analysis. The combination of these phases resulted in morphological and structural heterogeneity, as observed in SEM and FTIR analyses, further improving its photocatalytic activity.^17,18,39,55^ Furthermore, the superior photoactivity of ZnO 100 may be enhanced by its larger surface area compared to other samples. Thus, the improved photocatalytic activity of this sample can be attributed to a combination of three parameters: high surface hydroxylation, suitable electronic properties and a high SSA.^18,39,45,55,65,75,76^ The surface hydroxylation probably contributed to the superior performance of these samples, some authors attributed the formation of ^•^OH radical from the hydroxyl groups previously adsorbed on the sample’s surface.^11,18,39,45,55^ Regarding the charge separation mechanisms commonly discussed for conventional p–n heterojunctions such as ZnO/BiOI, the present system exhibits a fundamentally different interfacial architecture. In this case, the formation of a hydroxyl-rich Zn­(OH)_2_ surface layer on ZnO gives rise to a functional surface heterointerface rather than a classical semiconductor heterojunction. Although Zn­(OH)_2_ is electronically insulating and not a typical semiconductor, its wide effective band gap and high density of surface states induce a localized band bending and a potential gradient at the ZnO/Zn­(OH)_2_ interface.^77,78^ This interfacial energetic discontinuity promotes spatial charge separation by preferentially trapping photogenerated holes at surface hydroxyl groups, which act as highly reactive hole-accepting and oxidation sites, while modulating electron accumulation within the ZnO core. As a result, the localized interfacial electric potential suppresses electron–hole recombination by limiting back-transfer processes, thereby extending the lifetime of reactive charge carriers.^79^ The enhanced photocatalytic performance relative to bare ZnO is thus attributed to this synergistic combination of interfacial potential modulation and surface hole trapping, in good agreement with the trends observed in the scavenging experiments.^80–83^

It can be observed that increasing the synthesis temperature to 150 and 200 °C produced ZnO nanoparticles free of impurities, theoretically it should be beneficial for photocatalytic activity, but the results show that ZnO 150 and ZnO 200 samples exhibit a slight lower photocatalytic activity than the ZnO 100 sample. Although suitable morphology and an appropriate crystallite size generally favor photocatalytic efficiency, higher temperatures likely led to the removal of surface hydroxyl groups, negatively impacting performance.^18^ Thus, higher synthesis temperatures may reduce the material’s pollutant degradation efficiency.^18,33,73^ Additionally, a reduction in the SSA (<1 m^2^/g) was observed for samples prepared at ZnO 150 and ZnO 200, leading to a decrease in the photocatalytic efficiency of these semiconductors.^33,73,84^

The ZnO photocatalyst was subjected to four consecutive degradation cycles using RhB (Figure 8a). The stability tests were carried out over 4 consecutive experiments, each consisting of 4 h of photocatalytic operation, during which the material maintained high efficiency with no significant loss of performance, thereby demonstrating its durability across the evaluated cycles. Moreover, the XRD analyses conducted before and after the stability tests showed that the crystalline structure remained essentially unchanged, confirming that the material did not undergo structural degradation during the process (Figure S5). Consistent observations were also obtained from morphological analyses (Figure S7), whose results indicate preservation of the initial morphology, further reinforcing the stability of the photocatalyst. Furthermore, the consistent photocatalytic performance over repeated cycles indicates that ZnO exhibits high stability and durability, highlighting its potential for applications in long-term photocatalytic processes.

**Figure 8:**
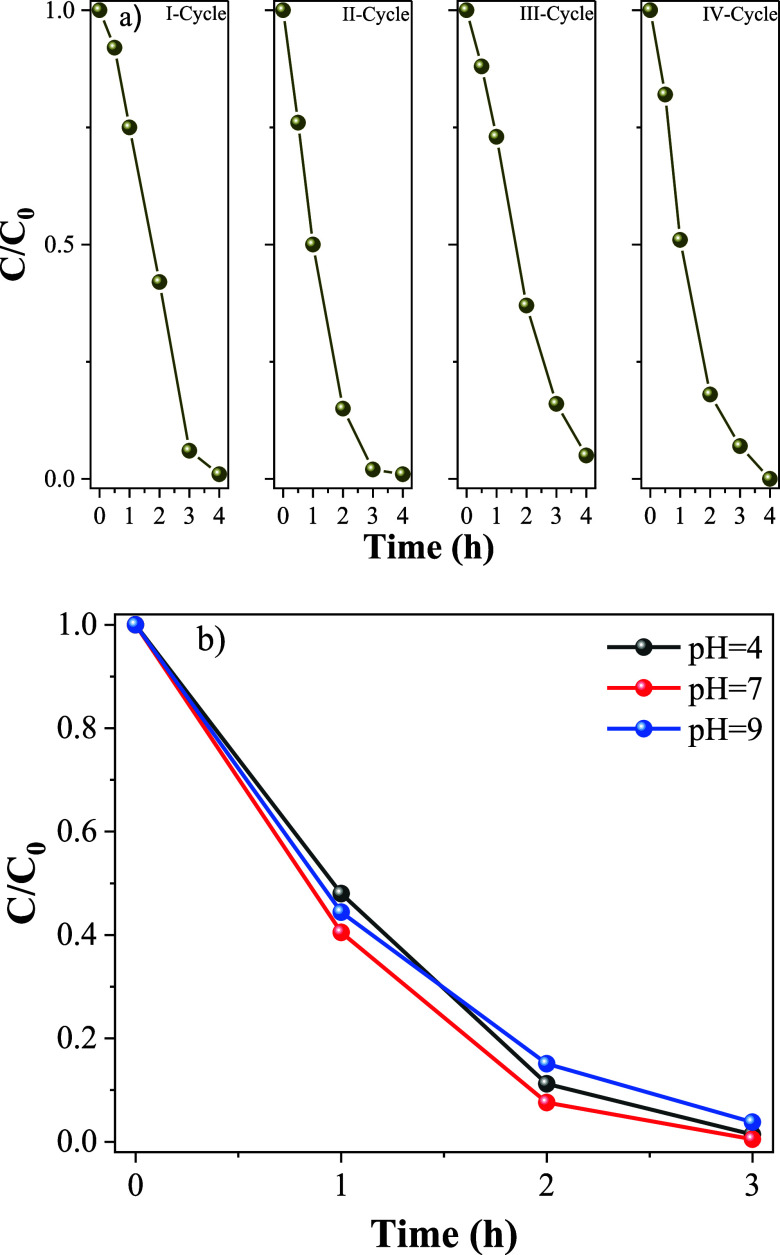
(a) Stability and reusability of the ZnO-100 catalyst evaluated over four consecutive cycles of RhB degradation, each cycle lasting 4 h. The results demonstrate the preserved catalytic activity of ZnO-100 upon repeated use, indicating good operational stability under the applied reaction conditions. (b) Photodegradation curves of RhB at different pH values using ZnO 100 over 3 h. No significant differences were observed among the three studied pH values, indicating that ZnO exhibited photocatalytic activity independent of pH.

COD analyses were performed after each degradation cycle (cycles 1 to 4) to estimate the remaining organic matter in solution (Table 2). The initial concentrations of the pollutants were 5 ppm for RhB, corresponding to COD values of 1080 mg L^–1^. After the completion of each degradation cycle. COD values decreasing from 1080 mg L^–1^ to a range between 133 mg L^–1^ and 27 mg L^–1^, corresponding to an overall reduction of approximately 97.5%. Overall, the COD results demonstrate the high efficiency of the proposed catalyst, ZnO 100, achieving substantial mineralization of both pollutants relative to their initial organic load.

**Table 2:** COD Values Measured after Each Degradation Cycle for AML and RhB

	COD in each cycle (mg L^–1^)			
pollutant	1°	2°	3°	4°
RhB	133	87	87	27

The influence of pH in the wastewater treatment is a crucial step to demonstrate the catalytic robustness of the ZnO samples and their practical applicability under conditions representative of real wastewater systems.^85^ Therefore, the photocatalytic degradation of RhB was evaluated under different pH conditions (4, 7, and 9), which covers the typical range of real wastewater (Figure 8b). ZnO 100 sample exhibited practically identical degradation efficiency in every pH evaluated. This suggests that, for the photocatalyst used, the interaction between the material surface and RhB molecules is relatively independent of pH within the studied range, it means ZnO 100 sample is robust and effective without the need for preacidification or alkalization of the effluent. However, while pH is not a limiting factor, the presence of scavenging ions in complex matrices remains a very complex factor to be investigated in future studies.^86^

To investigate the general degradation mechanism of the tested samples, hydroxyl radical detection was carried out using terephthalic acid as a probe molecule. This method enabled a more precise assessment of the materials’ ability to generate hydroxyl radicals.^87^ The results (Figure S7) show that all ZnO samples synthesized via hydrothermal treatment (100–200 °C) exhibited similar ^•^OH radical generation rates, all of which were significantly higher than that of the Prec sample Table 3. This suggests that hydroxyl radical formation (Figure 9a) plays some role in the observed photocatalytic activity. However, since the ZnO 100 sample showed superior performance in the degradation of all tested pollutants despite generating similar ^•^OH levels to the other ZnO samples, it is likely that additional reactive species, such as superoxide radicals (^•^O_2_^–^), also contribute significantly to the degradation mechanism.^88^ Therefore, the photocatalytic efficiency did not follow the same trend, and the ZnO 100 sample outperformed the others, likely due to the optimized combination of its structural properties, such as the larger surface area, which promotes adsorption and efficient charge separation.

**Table 3:** Generation Rates of Hydroxyl Radicals Measured Indirectly by TPA Probe Fluorescence Catalyzed by the ZnO Samples

sample	*k*_obs_ (OH^•^) (10^3^ min^–1^)
Prec	1.60
ZnO 100	15.39
ZnO 150	16.24
ZnO 200	17.25
control experiment	0.020

**Figure 9:**
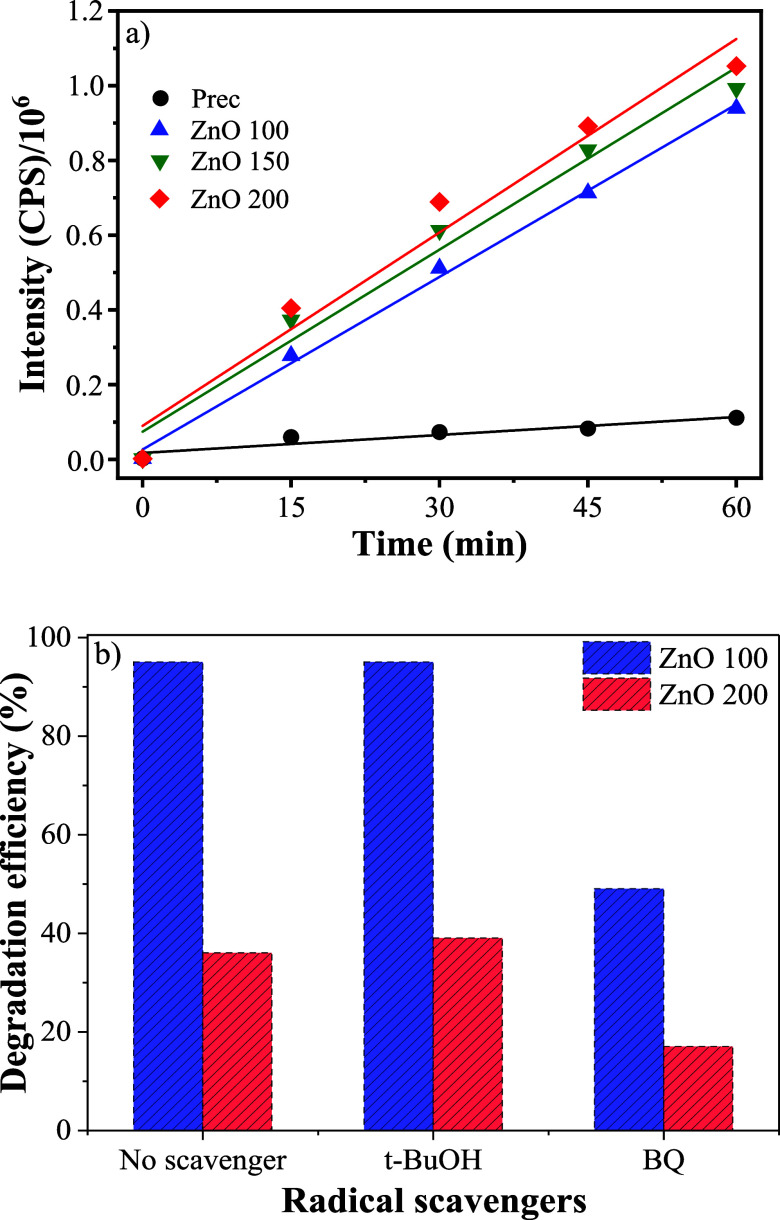
(a) Formation rates of hydroxyl radicals measured indirectly by TPA probe fluorescence catalyzed by the ZnO samples. (b) Effect of radical scavengers (*t*-BuOH and BQ) on the photocatalytic degradation efficiency of RhB by ZnO 100 after 240 min of irradiation.

To elucidate the photocatalytic mechanism and identify the primary reactive species involved in the RhB degradation over ZnO 100 and ZnO 200 samples, trapping experiments were conducted. *tert*-Butyl alcohol (*t*-BuOH) and benzoquinone (BQ) were introduced as specific scavengers for hydroxyl radicals (^•^OH) and superoxide radicals (^•^O_2_^–^), respectively.^40^ Figure 9b, presents comparative degradation efficiency after 240 min of irradiation in the presence of these scavengers. The results indicate that the addition of BQ significantly suppressed the photocatalytic activity of both ZnO samples, causing an approximate 50% reduction in degradation efficiency compared to the control test. Conversely, the presence of *t*-BuOH exhibited a negligible inhibitory effect on the degradation process for both samples.

The distinct behaviors observed in the scavenging experiments confirms that ^•^O_2_^–^ play a pivotal role in the degradation mechanism for both ZnO 100 and ZnO 200 samples. The limited impact of *t*-BuOH suggests that bulk hydroxyl radicals are not the predominant oxidizing species in this system. Furthermore, the fact that BQ inhibition was incomplete (reaching only ∼50%) implies the substantial contribution of another active species. This is attributed to direct oxidation via photogenerated valence band holes acting on the adsorbed dye molecules, a pathway not readily inhibited by the selected scavengers in the bulk solution.

Therefore, we can conclude that photogenerated electrons in the conduction band react with adsorbed oxygen to produce the highly active ^•^O_2_^–^ radicals. Simultaneously, the photogenerated holes in the valence band directly oxidize the RhB molecules adsorbed on the catalyst surface. Therefore, the synergistic action of ^•^O_2_^–^ radicals and direct h^+^ oxidation is responsible for the efficient mineralization of RhB by both ZnO 100 and ZnO 200 catalysts.

## 4 Conclusions

The ZnO nanoparticles were successfully synthesized under different hydrothermal conditions, resulting in a hexagonal crystalline structure of the wurtzite type. ZnO samples showed photocatalytic activity in the degradation of different organic pollutants kinds (dyes and drugs), demonstrating the versatility of the ZnO photocatalysts. The sample treated at 100 °C stands out as the most effective for pollutant degradation, mainly due to its larger SSA and rod-like morphology. In addition, this sample presented an interface between the Zn­(OH)_2_ and ZnO, which can increase the charge carrier lifetime. ZnO 100 sample exhibited a high stability under four cycles of reuse. Through fluorescence analysis and scavenger experiments, it was confirmed that all the obtained samples exhibited reactive species generation capabilities. These analyses demonstrated that the superoxide radical is the main reactive species responsible for the degradation of organic pollutants. Furthermore, the predominance of an indirect reaction mechanism helps explain the high efficiency of the ZnO-100 sample, which remained essentially unaffected by the evaluated pH range, highlighting its robustness under different reaction conditions. Thus, the proposed synthesis method proved to be efficient in obtaining ZnO nanorods, allowing precise control of the physicochemical properties by varying the synthesis parameters.

## Supplementary Material



## Notes

Photoreactor configuration and experimental setup; schematic of terephthalic acid oxidation for hydroxyl radical detection; crystallite size analysis from XRD (Scherrer equation); particle size distribution from SEM images; UV–vis diffuse reflectance spectra and band gap estimation (Kubelka–Munk method); structural and morphological stability before and after photocatalytic cycles (XRD and SEM); and fluorescence probe raw data (PDF)
