# Supplementary material for: Hydrothermal Synthesis of High-Performance ZnO Nanorods for Enhanced Photocatalytic Degradation of Organic Pollutants
Source: ACS Omega. 2026 Apr 14;11(16):24725–38. doi: 10.1021/acsomega.6c01314 (PMC13130113; doi:10.1021/acsomega.6c01314)
Supplement: Supplementary file 1 [file ao6c01314_si_001.pdf]

SUPPORTING INFORMATION

Hydrothermal synthesis of high-performance ZnO nanorods for enhanced photocatalytic degradation of organic pollutants

Gonçalves J. Marrenjo[1,2], Paulo H. H. Nunes[1], Karen K. L. Augusto[1], Jéssica C. Almeida[4], Caue Ribeiro[4], Antônio O. T. Patrocínio[1,3], Osmando F. Lopes[1*]

[1] Laboratoty of Photochemistry and Materials Science, Institute of Chemistry, Federal University of Uberlândia, Avenida João Naves de Avila, 2121, 34800-902 Uberlândia, Minas Gerais, Brazil

[2] Save University, Department of Natural Sciences, FPLM Avenida FPLM, 111, +25829371110, Massinga, Inhambane, Mozambique.

[3] Centro de Excelência em Hidrogênio e Tecnologias Energéticas Sustentáveis (CEHTES) Federal University of Goias, Goiânia, Goiás, Brazil

[4]EMBRAPA Instrumentação– Rua XV de Novembro, 1452– CP 741, CEP 13560-970 São Carlos, SP, Brazil

*e-mail: osmando@ufu.br

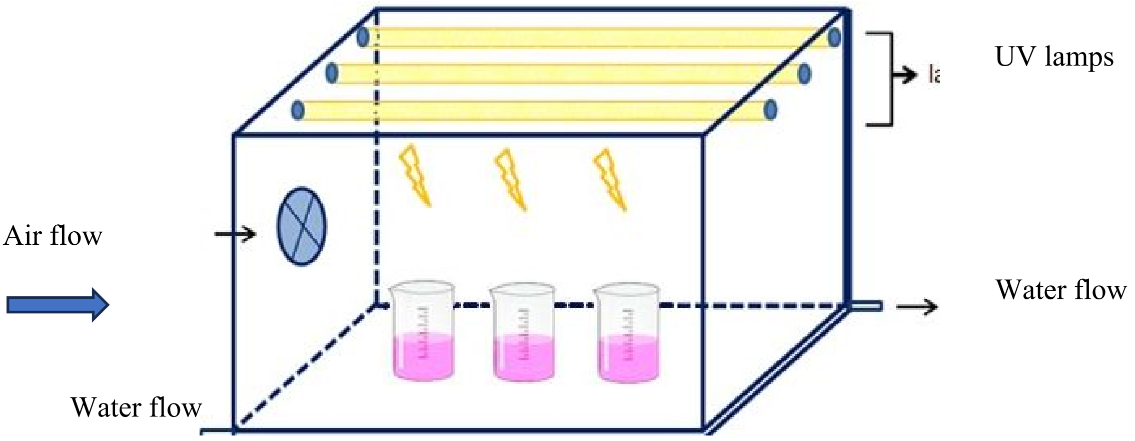

Figure S1. Photoreactor used in the experimental procedure for pollutant degradation, equipped with 15 W Philips TUV UVC lamps (maximum intensity at 254 nm, average irradiance of 40 W m^{-2}), and operated at a controlled temperature of 18 °C.

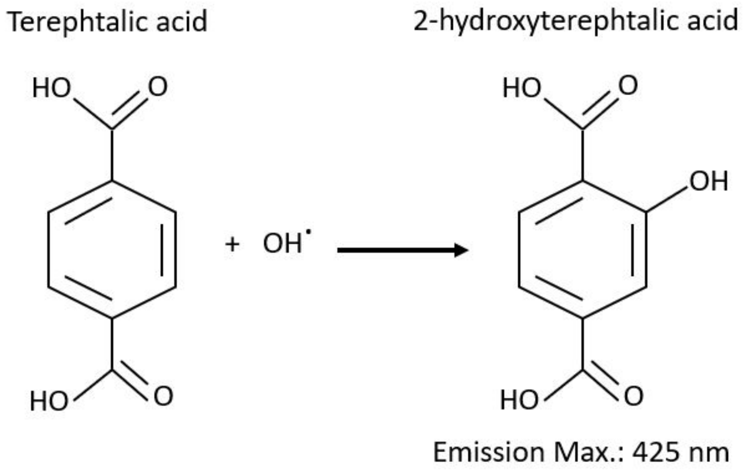

Figure S2. General depiction of the oxidation of terephtalic acid by hydroxyl radicals and formation of 2-hydroxyterephtalic acid (a fluorescent probe).

Table S1. Crystallite size (D) of ZnO samples treated at different temperatures calculated by Scherrer equation using XRD data.

Samples	Xc	Wc	D (nm)
ZnO 100	52.591	0.2646	34.898
ZnO 150	52.5826	0.300	31.444
ZnO 200	52.656	0.305	30.857

Table S2: Particle size distribution (L × W) of precursor and ZnO samples based on SEM images

Sample	**Average size (L×W)** µm
SEM: Prec	(14.6±1.5 × 14.1±2.8) µm
SEM: ZnO 100	(3.4±0.9 × 0.5±0.1) µm
SEM: ZnO 150	(3.4±0.5 × 0.6±0.1) µm
SEM: ZnO 200	(4.0±1.1 × 0.8±0.2) µm

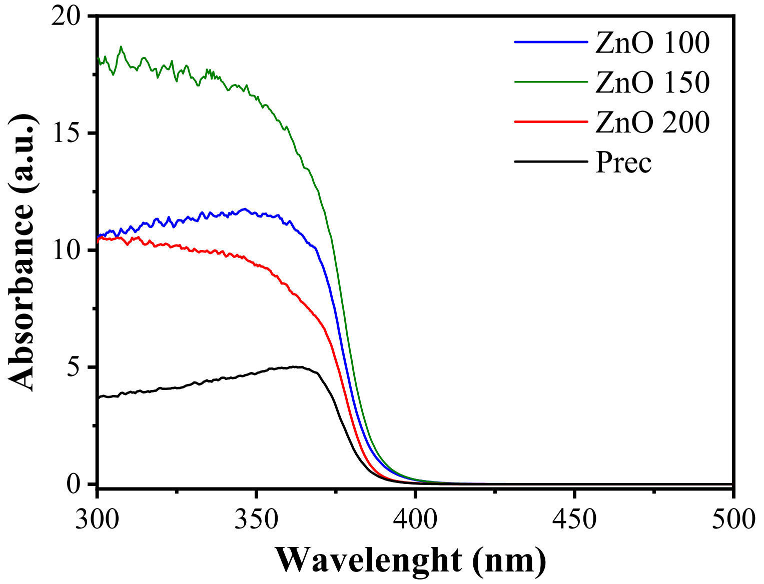

Figure S3. UV-Vis DRS analysis of the ZnO samples. All samples exhibited absorption around 380 nm, indicating their sensitivity to ultraviolet radiation.

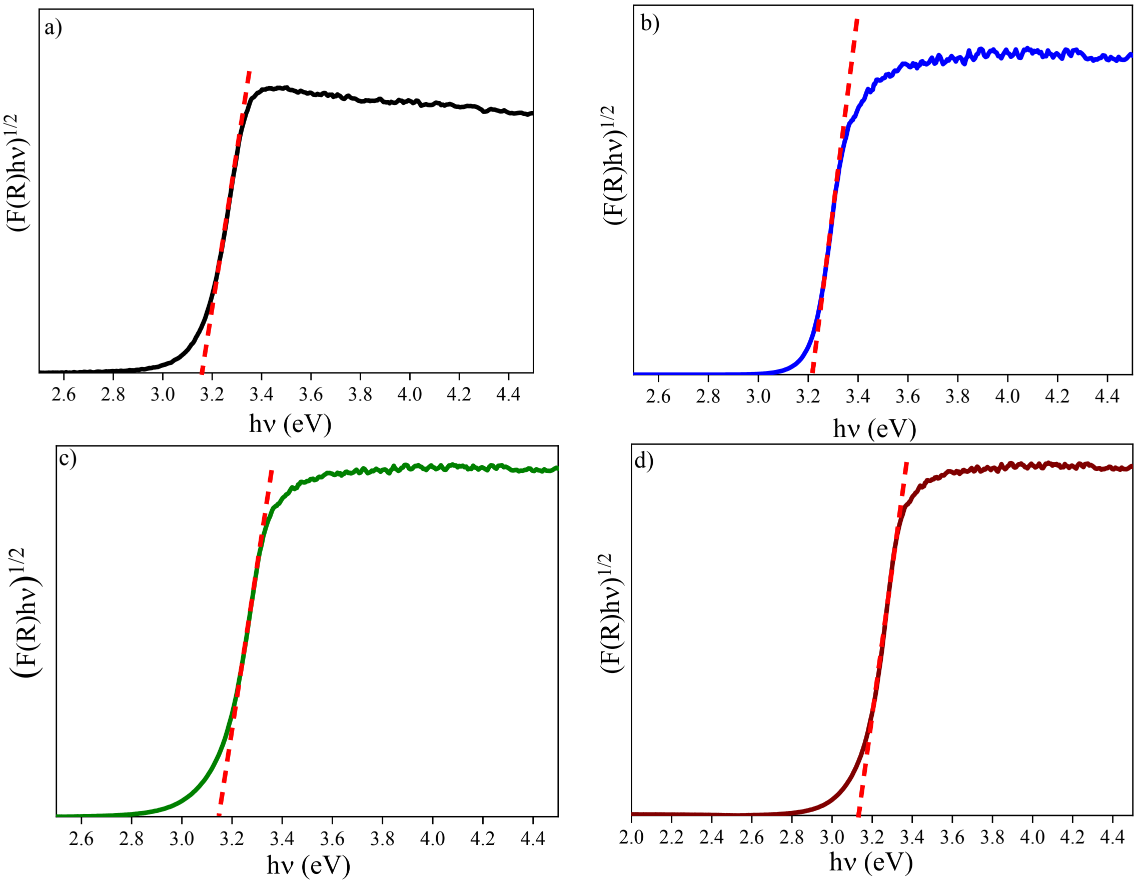

Figure S4. The evolution of the band gap value was evaluated using the Kubelka–Munk method for a) Prec, b) ZnO-100, c) ZnO-150 and d) ZnO-200 samples, and all samples exhibited a band gap of approximately 3.2 eV.

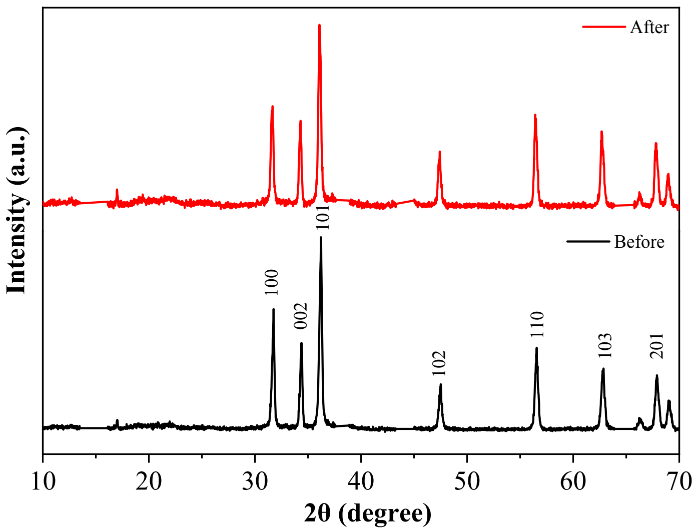

Figure S5. XRD patterns of ZnO before (a) and after the reaction (b): the material remained structurally stable, with no significant changes in the diffraction peaks, indicating that even after four reaction cycles, ZnO preserved its crystalline structure and crystallographic phase.

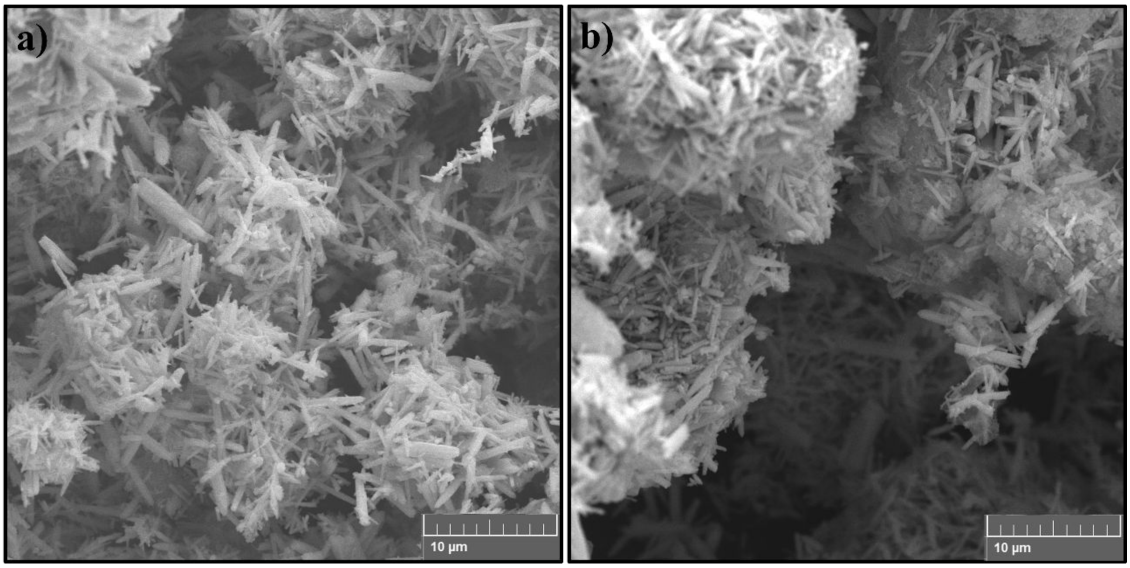

Figure S6. Morphology of ZnO before (a) and after the reaction (b): the material remained stable, with no significant changes, indicating that even after four reaction cycles, ZnO preserved its morphological integrity

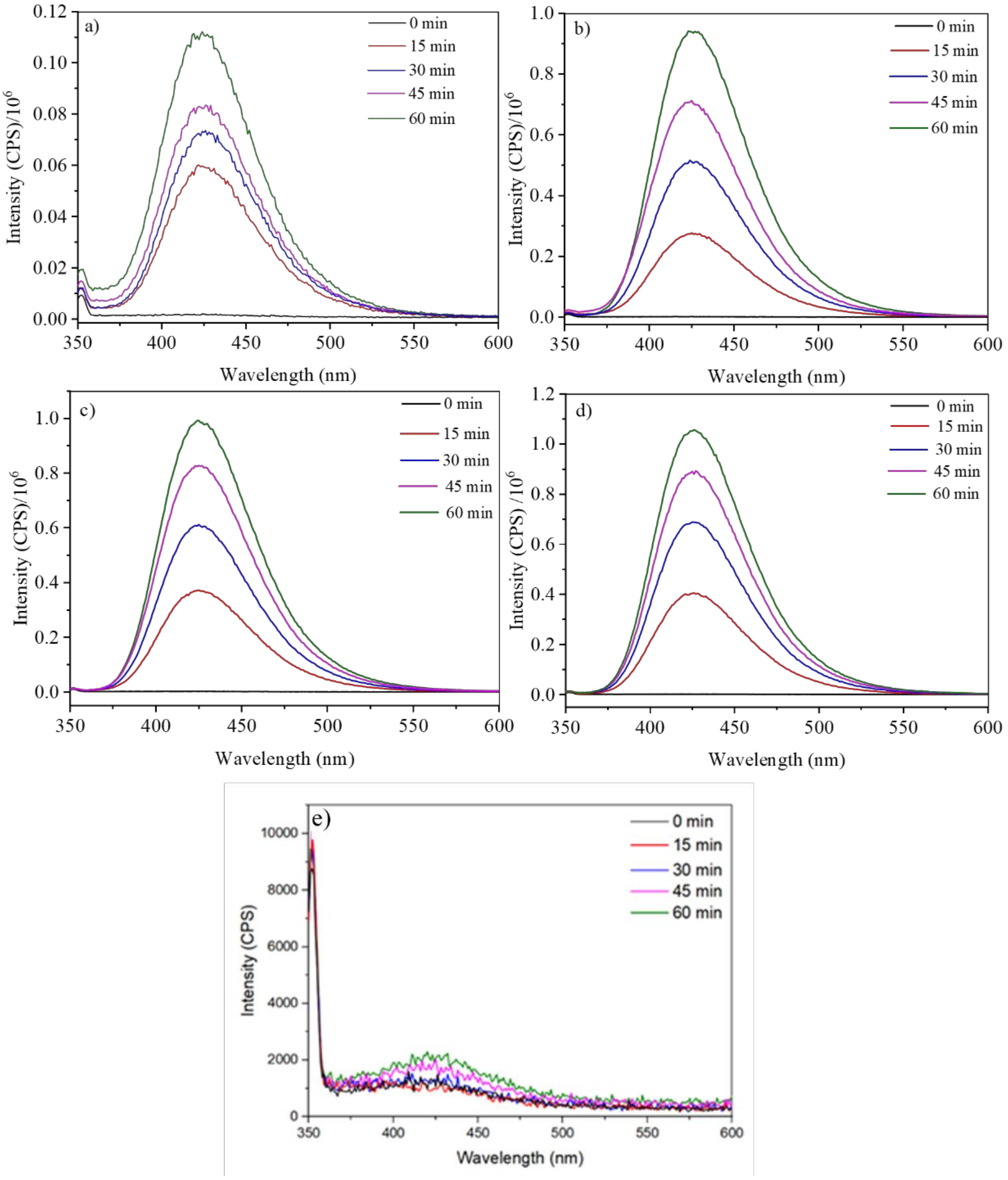

Figure S7: Raw data obtained from the fluorescence probing with terephtalic acid for (a) Prec, (b) ZnO 100, (c) ZnO 150, (d) ZnO 200 samples and (e) control experiment without photocatalyst.
